# Impact of Adjuvant Use of Midodrine to Intravenous Vasopressors: A Systematic Review and Meta-Analysis

**DOI:** 10.1155/2021/5588483

**Published:** 2021-05-15

**Authors:** Ahmad Al-Abdouh, Sadam Haddadin, Atul Matta, Ahmad Jabri, Mahmoud Barbarawi, Waiel Abusnina, Qais Radideh, Mohammed Mhanna, Dante A. Suffredini, Erin D. Michos

**Affiliations:** ^1^Department of Medicine, Ascension Saint Agnes Hospital, Baltimore, MD, USA; ^2^Department of Pulmonary, Critical Care and Sleep Medicine, Einstein Medical Center, Philadelphia, PA, USA; ^3^Department of Cardiology, MetroHealth Medical Center, Cleveland, OH, USA; ^4^Department of Cardiology, University of Connecticut, Farmington, Mansfield, CT, USA; ^5^Department of Cardiology, Creighton University School of Medicine, Omaha, NE, USA; ^6^Midwest Cardiovascular Research Foundation, Davenport, IA, USA; ^7^Department of Medicine, University of Toledo, Toledo, OH, USA; ^8^Section of Critical Care, Department of Medicine, Ascension Saint Agnes Hospital, Baltimore, MD, USA; ^9^Division of Cardiology, Johns Hopkins University School of Medicine, Baltimore, MD, USA

## Abstract

**Purpose:**

To evaluate the efficacy and safety of midodrine use in intensive care units (ICU) to facilitate weaning off intravenous vasopressors (IVV).

**Methods:**

We searched PubMed/MEDLINE, Cochrane library, and Google Scholar (inception through October 18^th^, 2020) for studies evaluating adjuvant use of midodrine to IVV in the ICU. The outcomes of interest were ICU length of stay (LOS), hospital LOS, mortality, IVV reinstitution, ICU readmission, and bradycardia. Estimates were pooled using the random-effects model. We reported effect sizes as standardized mean difference (SMD) for continuous outcomes and risk ratios (RRs) for other outcomes with a 95% confidence interval (CI).

**Results:**

A total of 6 studies were found that met inclusion criteria and had sufficient data for our quantitative analysis (1 randomized controlled trial and 5 retrospective studies). A total of 2,857 patients were included: 600 in the midodrine group and 2,257 patients in the control group. Midodrine use was not associated with a significant difference in ICU LOS (SMD 0.16 days; 95% CI −0.23 to 0.55), hospital LOS (SMD 0.03 days; 95% CI −0.33 to 0.0.39), mortality (RR 0.87; 95% CI 0.52 to 1.46), IVV reinstitution (RR 0.47; 95% CI 0.17 to 1.3), or ICU readmission (RR 1.03; 95% CI 0.71 to 1.49) when compared to using only IVV. However, there were higher trends of bradycardia with midodrine use that did not reach significance (RR 7.64; 95% CI 0.23 to 256.42).

**Conclusion:**

This meta-analysis suggests that midodrine was not associated with a significant decrease in ICU LOS, hospital LOS, mortality, or ICU readmissions.

## 1. Introduction

Intravenous vasopressors (IVVs) are an essential component in the management of shock following adequate fluid resuscitation [1, 2]. Various guidelines are available for the management of vasopressor therapy to achieve the clinically indicated blood pressure goals [3, 4]. However, in some cases, discontinuation of IVV can be challenging due to continued vasoplegia [5]. In these cases, continuous need for IVV therapy may prevent patients from being discharged from the intensive care unit (ICU) due to the need for close hemodynamic monitoring and in turn may prolong their hospital length of stay [6]. Midodrine, an oral alpha-1 agonist, was originally approved by U.S. Food and Drug Administration (FDA) for the treatment of orthostatic hypotension. However, it has been used as an off-label agent in the management of several other conditions such as dialysis-induced hypotension [7], hepatorenal syndrome [8], neurogenic hypotension [9], and hypotension associated with carotid artery stenting [10]. There has also been an increasing trend towards using midodrine as an adjunctive agent in an attempt to weaning IVV in patients with resolving shock and in turn reducing their ICU and hospital length of stay (LOS) [11]. Midodrine has several side effects including bradycardia, hypertension, ischemia, gastrointestinal upset, piloerection, pruritus, and dysuria that may limit its safety [12, 13].

Various observational studies have been done evaluating the effect of midodrine to facilitate IVV weaning [13–20]. Recently, the results from the “Effect of midodrine versus placebo on time to vasopressor discontinuation in patients with persistent hypotension in the intensive care unit (MIDAS) - an international randomized controlled trial (RCT)” [6] have also been published. We performed a systematic review and meta-analysis to evaluate the effects of midodrine use in critically ill patients recovering from the shock.

## 2. Methods

### 2.1. Search Strategy

**Figure 1:**
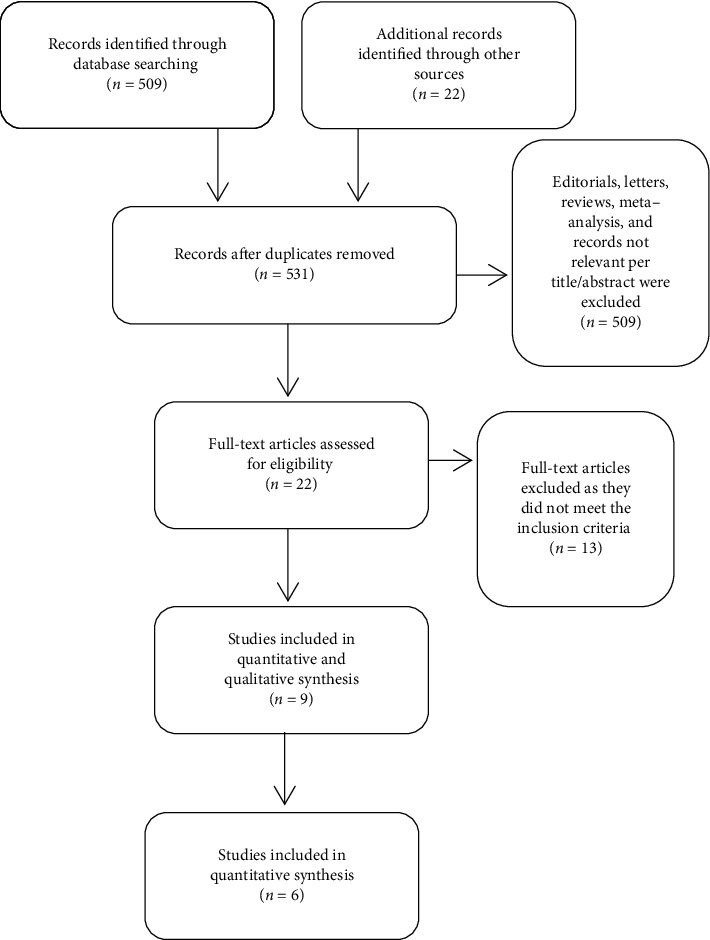
Details of the search results.

This meta-analysis was conducted in accordance with Cochrane collaboration guidelines [21] and reported according to the Preferred Reporting Items for Systematic Review and Meta-Analysis (PRISMA) protocols [22]. A literature search was performed using electronic databases of PubMed/MEDLINE, Cochrane reviews, and Google scholar databases (from inception through October 18^th^, 2020), without language limitations, by two independent reviewers (AA and SH). Any discrepancy was resolved by a third reviewer (MB). The following keywords were used: “midodrine”; “vasopressor”; “shock”; “intensive care unit”; and “critical care.” References of retrieved studies were screened for further relevant studies suitable for this meta-analysis (Figure 1).

### 2.2. Study Selection

Our analysis included prospective and retrospective studies that compared using midodrine with IVV versus IVV only in patients admitted to ICU with shock. RCTs, case-control, and cohort studies were eligible for inclusion. Reviews and editorials were excluded.

### 2.3. Data Abstraction and Quality Assessment

The data abstraction was performed on a prespecified data collection form by two independent reviewers (AA and SH), and any discrepancy was resolved by a third reviewer (AJ).

### 2.4. Outcomes of Interest

Abstracted data included IVV duration, ICU LOS, hospital LOS, mortality, IVV reinstitution, ICU readmission, and bradycardia.

### 2.5. Statistical Analysis

When unavailable, the means and standard deviations were calculated from the median and interquartile ranges that were provided in the selected studies as described by Wan et al. [23]. Estimates were pooled using a random-effects Mantel–Haenszel model. The DerSimonian and Laird method was used for estimation of *τ*^2^. We reported effect sizes as standardized mean difference (SMD) for ICU length of stay, hospital length of stay, and vasopressors duration. We reported risk ratios (RRs) for other outcomes. All effect sizes were reported with a 95% confidence interval (CI). The 95% CIs that did not cross 1 were considered statistically significant. We used *I*^2^ statistics to measure the extent of unexplained statistical heterogeneity: *I*^2^ greater than 50% was considered a high degree of between-study statistical heterogeneity. Analyses were performed using R studio.

### 2.6. Heterogeneity Evaluation and Sensitivity Analysis

We evaluated heterogeneity using Baujat plots to determine the studies with the most contribution to heterogeneity and then repeating the analyses after excluding them. Baujat plot is a graphical method that can identify the studies that are a source of heterogeneity. It is a two-dimensional graph with the *x*-axis representing the contribution of the study to heterogeneity and the *y*-axis representing the influence of the trial on the overall effect [24]. We also performed leave-one-out analyses by excluding one study each time and then repeated the analyses to detect any influential effect of any of the included studies.

### 2.7. Quality Assessment

The quality of the included studies was assessed using the Newcastle–Ottawa Scale for observational studies [25] and the Revised Cochrane risk-of-bias (RoB 2) tool for the RCT [21]. Two authors (AA and SH) assessed each study independently for bias. We resolved discrepancies by consensus. Publication bias was not evaluated due to the limited number of the included studies [26].

## 3. Results

### 3.1. Summary of Studies

A total of 845 articles were obtained from a comprehensive electronic database search. After a thorough review, we found 9 studies (one RCT and 8 retrospective studies) that investigated midodrine and IVV use versus using only IVV in ICU settings. Five retrospective studies were only published in abstract form, and 3 of them were not included in our quantitative analysis due to lack of enough data. The search process is detailed in Figure 1. The pertinent details of the included studies are illustrated in Table 1.

A total of 2,857 patients were included in our analysis from 1 RCT and 5 retrospective studies: 600 patients in the midodrine and IVV group and 2,257 patients in the IVV only group. The baseline characteristics of patients included in our study are detailed in Table 2.

### 3.2. Outcomes

**Figure 2:**
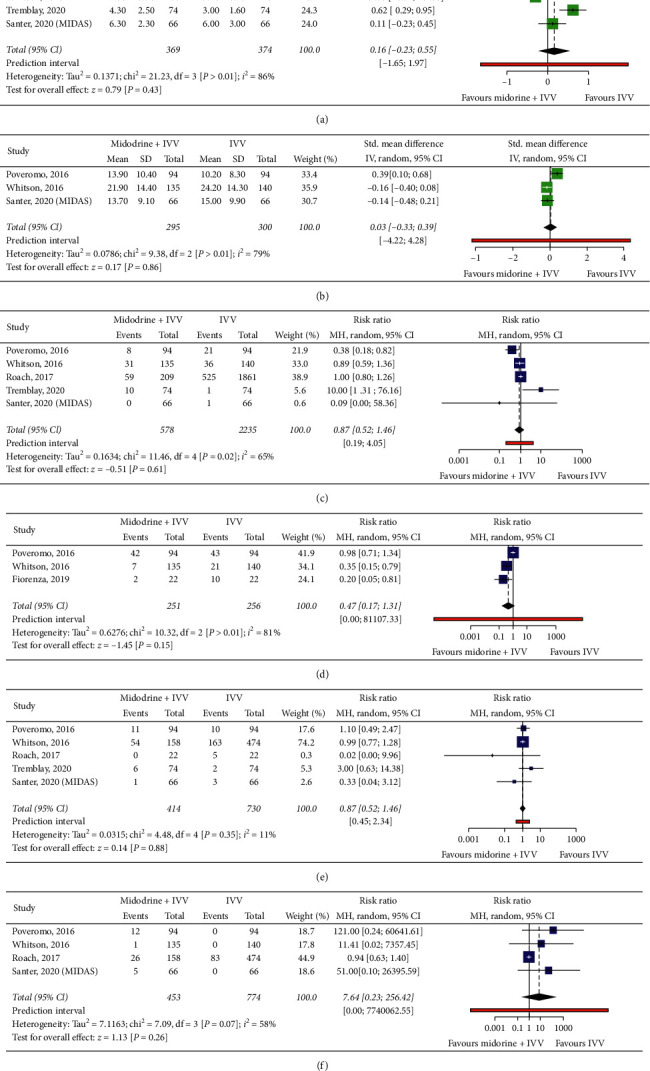
(a) Forest plot of mean duration of ICU length of stay. (b) Forest plot of mean duration of hospital length of stay. (c) Forest plot of in-hospital mortality. (d) Forest plot of intravenous vasopressors reinstitutions. (e) Forest plot of ICU readmission. (f) Forest plot of bradycardia.

Midodrine use along with IVV was not associated with the significant difference in ICU LOS (SMD 0.16 day; 95% CI −0.23 to 0.55; *p*=0.43; *I*^2^ = 86%) (Figure 2(a)) or hospital LOS (SMD 0.03 days; 95% CI -0.33 to 0.0.39; *p*=0.86; *I*^2^ = 79%) (Figure 2(b)) when comparing to using only IVV. Midodrine was not associated with significant decrease in mortality (RR 0.87; 95% CI 0.52 to 1.46; *p*=0.61; *I*^2^ = 65%), IVV reinstitution (RR 0.47; 95% CI 0.17 to 1.31; *p*=0.15; *I*^2^ = 81%), or ICU readmission (RR 1.03; 95% CI 0.71 to 1.49; *p*=0.88; *I*^2^ = 11%) when compared to using only IVV (Figures 2(c)–2(e)). However, there were higher trends of bradycardia in the midodrine group that did not reach significance (RR 7.64; 95% CI 0.23 to 256.42; *p*=0.26; *I*^2^ = 58%) (Figure 2(f)).

### 3.3. Heterogeneity Evaluation and Sensitivity Analyses

Heterogeneity was noted to be high in most of our studied outcomes. Baujat plots were done to evaluate this heterogeneity. Leave-one-out analyses were done to detect any influential effects of the included studies, especially those with the highest contribution to heterogeneity.

1. ICU LOS: Whitson et al. [13] was found to be the study with the highest contribution to heterogeneity and was the only study including only patients with septic shock in this outcome. After excluding it, the *I*^2^ dropped from 86% to 58%, and the SMD of ICU LOS became significant, favoring the control group (SMD 0.32 days; 95% CI 0.04 to 0.61; *I*^2^ = 58%) (Supplementary Figures 1A and 2A).
2. Hospital LOS: Poveromo et al. [20] was the study with the highest contribution to heterogeneity and by excluding it, the *I*^2^ dropped from 79% to 0%. However, this did not lead to any significant changes in the SMD of hospital LOS (SMD −0.15 days; 95% CI −0.35 to 0.04; *I*^2^ = 0%) (Supplementary Figures 1B and 2B).
3. Mortality: Poveromo et al. [20] was also the study with the highest contribution to heterogeneity. When we excluded it, the *I*^2^ dropped from 65% to 49%. Leave-one-out analyses of the studies evaluating this outcome did not show any significant changes in the results (Supplementary Figures 1C and 2C).
4. IVV reinstitution: three studies assessed this outcome; both Fiorenza et al. [19] and Whitson et al. [13] showed a significant decrease of IVV reinstitution while Poveromo et al. [20] did not show a significant difference. Although Baujat plot showed that Whitson et al. [13] contributed most to heterogeneity; the lowest possible heterogeneity can be achieved by excluding Poveromo et al. [20] as it showed different results from the others; this dropped the *I*^2^ from 81% to 0%, and repeating the analysis after excluding it showed the midodrine group now had significantly lower rates of IVV reinstitution (RR 0.47; 95% CI 0.15 to 0.61; *I*^2^ = 0%) (Supplementary Figures 1D and 2D).
5. ICU readmissions: the heterogeneity was already low in this outcome. However, excluding Tremblay et al. [16] or Fiorenza et al. [19] dropped *I*^2^ from 11% to 0%. Leave-one-out analyses of the studies evaluating this outcome did not show any significant changes in the results (Supplementary Figures 1E and 2E).
6. Bradycardia: Roach et al. [18] contributed the most to heterogeneity and excluding it dropped the *I*^2^ from 58% to 0%; furthermore, this made bradycardia significantly higher among patients in the midodrine group but with a very long 95% CI (RR 42.68; 95% CI 1.12 to 1629.53; *I*^2^ = 0%) (Supplementary Figures 1E and 2E).

### 3.4. Quality Assessment

We assessed the quality of the included studies using the Newcastle–Ottawa Scale for cohort studies [25] and the Revised Cochrane risk-of-bias tool for randomized controlled trials [21], as shown in Supplementary Table 1. For Newcastle–Ottawa Scale, each asterisk counts as one point. The maximum points are two for comparability and one for all other categories (Supplementary Table 1). Each star adds to the total score. A score of less than five is considered low quality, five to six is medium quality, while seven to nine is high quality. All the included studies scored moderate to high in quality assessment in our meta-analysis.

## 4. Discussion

Based on our meta-analysis, the addition of midodrine had no effect on the ICU LOS, hospital LOS, mortality, ICU readmission, or IVV reinstitution. To the best of our knowledge, this is the first meta-analysis that includes the data from the MIDAS trial [6] and Tremblay et al. [16] study.

Continued IVV administration without any signs of organ hypoperfusion may prolong recovery from shock and can lead to prolonged ICU and hospital LOS. Midodrine, an oral alpha-1 agonist, has been increasingly used in patients recovering from shock to facilitate the weaning of IVV and in turn reducing their hospital length of stay, [11] but our findings provide supportive evidence that this may not be a beneficial strategy or even cause harm.

Previous retrospective studies and one RCT have been conducted with conflicting results; some showed that midodrine is helpful in this process [13, 14, 17, 19], and others including the RCT showed no benefit of adding midodrine to facilitate weaning off IVV [6, 15, 18]. Surprisingly, two studies showed that midodrine increases hospital and ICU LOS [16, 20]. Based on these conflicting results, our meta-analysis was performed to solve this dilemma as new studies have been published after the last meta-analysis by Hammond et al. [27].

Among the prior studies reviewed, Whitson et al. [13], Hailu et al. [17], Liu et al. [14], and Nadhim et al. [15] were the only ones that included patients with septic shock. Whitson et al. [13], Liu et al. [14] (abstract), and Hailu et al. [17] (abstract) showed a significant reduction in IVV duration, ICU LOS, and Hospital LOS while Nadhim et al. [15] (abstract) did not show a significant decrease in IVV duration. Analysis limited to studies that included patients with septic shock was not done as 3 of these studies are just published as abstracts and did not report standard deviations for means or interquartile ranges for medians to pool them in our quantitative analysis.

Santer et al. [6] (MIDAS trial) showed no significant difference between the two groups in terms of time to discontinuation of IVV (difference +1 day; 95% CI −10.4 to 12.3; *p*=0.62); ICU LOS (difference 0; 95% CI −0.5 to 0.5 days; *p*=0.46), and hospital length of stay (difference −3 days; 95% CI −6.3 to 0.3; *p*=0.46). Though this was a well-designed trial, it had several limitations including a small sample size and a heterogeneous population. The majority of patients in both groups were postoperative/surgical (68.2% in treatment arm and 63.6% in control arm) and only 19.7% of patients had sepsis so this makes the results less applicable to septic patients where midodrine is mostly used in clinical practice based on previously mentioned retrospective studies.

Similar heterogeneity has been observed in other retrospective studies and this might have resulted in variability in the available outcomes. Poveromo et al. [20] suggested that midodrine can be used as an adjunctive treatment in the weaning of IVV in patients with resolving shock as the median time to discontinue IVV after starting midodrine was 1.2 days (IQR 0.5–2.8) but this outcome was not compared to the control group. That study showed that ICU discharge happened sooner after IVV discontinuation in the midodrine group and based on that concluded possible benefit of midodrine in this process, though hospital LOS was longer in the midodrine group and ICU LOS or readmissions were not different between both groups. They included patients with various etiologies of shock but the majority of their patients in both the groups were from a cardiovascular ICU. Also, the patients who received midodrine had a significantly lower median Acute Physiology and Chronic Health Evaluation (APACHE) IV score (*p*=0.02) and a significantly higher incidence of corticosteroid administration during hospitalization (*p*=0.04) which could affect the results. Roach et al. [18] (abstract) also had more patients with septic shock in the midodrine group (70.8% vs. 57.8%; *p* < 0.01). However, in their study midodrine failed to show a significant reduction in IVV duration, ICU LOS, or hospital LOS. This could have been contributed by the fact that they had sicker patients in the midodrine group as the APACHE III score was significantly higher among the midodrine group (84 vs. 77; *p* < 0.01). Fiorenza et al. [19] (abstract) was the only study to include just extubated patients who still require IVV (less than 15 mcg/hr of norepinephrine equivalent) with different etiologies; this study showed that midodrine decreased hospital LOS, ICU readmission, and IVV reinstitutions. Tremblay et al. [16] was the only study to include patients after cardiac surgery with cardiopulmonary bypass, and it showed increased ICU LOS and higher mortality among the midodrine group.

It is worth mentioning that IVV duration was not studied in our analysis as enough data were only reported in Santer et al. [6] trial which included the majority of surgical patients and Tremblay et al. study which included patients after cardiac surgery, and so this makes the results of this outcome inapplicable to others like septic patients.

Midodrine has several side effects, such as bradycardia, hypertension, ischemia, and gastrointestinal upset. Our meta-analysis is the first to evaluate the safety profile of midodrine. Bradycardia was the most commonly reported side effect in the included studies (reported in four studies); hence, we were able to evaluate it. Our analysis did not show a significant difference in bradycardia between midodrine and control groups. However, in a sensitivity analysis, when we excluded Roach et al. which contributes most to the heterogeneity, this made bradycardia significantly higher among the midodrine group but with exceptionally wide confidence interval and low precise estimates. We used the available unmatched data from Roach et al. and this could explain the heterogeneity contribution from this study.

We evaluated heterogeneity and tried to overcome that by doing leave-one-out analyses. Regarding ICU LOS, Whitson et al. [13], which included patients with septic shock, was the only study that showed benefit for midodrine; excluding the study by Whitson made the SMD in ICU LOS favor the control group. This may indicate that the benefit of midodrine that was evident in the population examined by Whitson et al. was offsetting the less favorable results in the other included studies before doing our sensitivity analysis. For IVV reinstitution, we found that excluding Poveromo et al. [20] (which included patients with various shock etiologies) and leaving Whitson et al. [13] (which included only patients with septic shock) and Fiorenza et al. [19] (which included only extubated patients but with varying shock etiologies) made IVV reinstitution significantly lower among the midodrine group. This result may emphasize the benefit of midodrine in weaning IVV and decreasing IVV reinstitution that is noted mainly in patients with septic shock and patients after extubation in this sensitivity analysis. For other outcomes, our sensitivity analyses did not lead to any change in the results.

Fortunately, further ongoing trials are evaluating midodrine use to wean off IVV in ICU, some are targeting specific population like septic patients (NCT03911817 and NCT03706053) and surgical patients (NCT01531959), while others are evaluating its use for all patients in ICU like LIBERATE trial (midodrine for the early liberation of vasopressor support in the ICU (NCT04489589). All these trials will help in creating guidelines for midodrine use in ICU to wean off IVV.

Our study has several limitations to be acknowledged. First, due to the availability of only one RCT, most of the involved studies are observational studies which increase the risk of selection bias and treatment bias [28]. Second, there is significant heterogeneity of the included patients and this can have considerable effects on outcomes. Third, the heterogeneity of most outcomes was high which could be due to heterogeneity of population between the included studies and the conflicting results among the included studies. However, we performed a detailed evaluation of heterogeneity by doing Baujat plots, and we did sensitivity analyses by excluding the studies with most contribution to heterogeneity in addition to performing leave-one-out analyses. Fourth, many studies were presented in abstract/poster form and we were unable to pool its results for some outcomes due to the inability to obtain data like standard deviations for means, interquartile ranges for medians, or adverse events like bradycardia. Fifth, means and standard deviations were calculated for some outcomes from medians and interquartile ranges and this assumes a normal distribution of the data which could be unrealistic.

## 5. Conclusion

In conclusion, midodrine use in patients requiring IVV in ICU was not associated with a significant decrease in ICU LOS, hospital LOS, mortality, IVV reinstitutions, or ICU readmissions. These results suggest the need for more RCTs to evaluate the role of midodrine in weaning off IVV.

## Figures and Tables

**Table 1:** Characteristics of the included studies.

Author	Type of study	Population	Duration of enrollment	Number of patients	Countries	Midodrine dose	Main results
Liu, 2010	Retrospective (abstract)	Patients requiring IVV for septic shock	December 2007 to December 2009	40	USA	—	Midodrine decreased the duration of IVV

Poveromo, 2016	Retrospective	Patients requiring IVV	January 2007 to March 2012	188	USA	10 mg every 8 hours (starting dose)	Midodrine increased hospital LOS and did not affect ICU LOS or ICU readmissions. Bradycardia occurred in 12.8% in midodrine group compared to 0% in control group

Whitson, 2016	Retrospective	Patients with septic shock requiring at least 24 h of IVV	November 2013 to November 2014	275	USA	10 mg every 8 hours (starting dose)	Midodrine decreased duration of IVV, reinstitution of IVV, and ICU LOS. No significant difference in bradycardia rates

Roach, 2017	Retrospective (abstract)	Patients requiring at least 7 days of IVV	September 2013 to September 2016	2070	USA	15 mg every 8 hours (starting dose)	Midodrine did not significantly decrease duration of IVV, hospital LOS, or ICU LOS. No significant difference in bradycardia rates

Fiorenza, 2019	Retrospective (abstract)	Patients who received less than 15 mcg/hr of norepinephrine equivalent after extubation	December 2016 to June 2018	44	USA	—	Midodrine decreased hospital LOS, ICU readmission, and vasopressors reinstitution

Nadhim, 2019	Retrospective (abstract)	Patients with septic shock requiring at least 24 h of IVV	January 2017 to March 2018	83	USA	—	Midodrine did not significantly decrease the duration of IVV

Hailu, 2020	Retrospective (abstract)	Patients with septic shock requiring at least 24 h of IVV	June 2013 to August 2018	166	USA	—	Midodrine decreased ICU LOS, hospital LOS, and IVV duration

Tremblay, 2020	Retrospective	Patients requiring IVV within the first week after cardiac surgery with cardiopulmonary bypass	January 2014 to January 2018	148	Canada	10 mg every 8 hours (starting dose)	Midodrine increased ICU LOS and was associated with higher mortality

Santer, 2020 (MIDAS)	Randomized controlled trial	Patients (adults) requiring single-agent IVV for more than 24 h	October 2012 to June 2019	132	USA and Australia	20 mg every 8 hours	Midodrine did not decrease time to IV vasopressors discontinuation and was associated with more bradycardia

ICU: intensive care unit; IVV: intravenous vasopressors; LOS: length of stay; MIDAS: effect of midodrine versus placebo on time to vasopressors discontinuation in patients with persistent hypotension in the intensive care unit.

**Table 2:** Demographics of participants of the included trials.

Study		Number	Age (SD)	Male (%)	(Mean ± SD) APACHE score	Corticosteroids administration
Liu, 2010	Midodrine	20	—	—	—	17 (85)
	Control	20	—	—	—	5 (25)

Poveromo, 2016	Midodrine	94	64.3 ± 15	64 (68.1)	61.3 ± 7.9 (APACHE 4)	52 (55.3)
	Control	94	65.9 ± 15.5	59 (62.8)	82 (66–93) (APACHE 4)	38 (40.4)

Whitson, 2016	Midodrine	135	69.3 ± 16	64 (47)	82.6 ± 26.4 (APACHE 4)	35 (26)
	Control	140	65 ± 19	79 (56)	84.3 ± 26.8 (APACHE 4)	40 (28.6)

Roach, 2017	Midodrine	158	—	—	84 (APACHE 3)	—
	Control	474	—	—	77 (APACHE 3)	—

Fiorenza, 2019	Midodrine	51	—	—	—	—
	Control	51	—	—	—	—

Nadhim, 2019	Midodrine	41	—	—	—	—
	Control	42	—	—	—	—

Hailu, 2020	Midodrine	83	—	—	—	—
	Control	83	—	—	—	—

Tremblay, 2020	Midodrine	74	68.3 ± 9.8	45 (60.8)	—	—
	Control	74	65.4 ± 11.5	47 (63.5)	—	—

Santer, 2020 (MIDAS)	Midodrine	66	70.0 ± 19.1	36 (54.4)	14.7 ± 7.9 (APACHE II)	—
	Control (placebo)	66	66.7 ± 22.3	32 (48.5)	14.8 ± 8.9 (APACHE II)	—

APACHE: acute physiology and chronic health evaluation; MIDAS: effect of midodrine versus placebo on time to vasopressors discontinuation in patients with persistent hypotension in the intensive care unit; SD: standard deviation.

## Data Availability

Data were abstracted from the included studies. The studies were included after a search that was done using electronic databases including PubMed/MEDLINE, Cochrane reviews, and Google scholar databases.

## Supplementary Materials

Supplementary Materials The supplementary material has detailed risk of bias and heterogeneity assessment, in addition to sensitivity analyses.
