# Supplementary material for: Impact of Adjuvant Use of Midodrine to Intravenous Vasopressors: A Systematic Review and Meta-Analysis
Source: Crit Care Res Pract. 2021 May 15;2021:5588483. doi: 10.1155/2021/5588483 (PMC8147551; doi:10.1155/2021/5588483)

**SUPPLEMENTARY APPENDIX**

| Title | Page |
| --- | --- |
| **Supplementary Table 1: The risk of bias assessment of the included studies** | 2 |
| **Supplementary Figure 1: Baujat plots of outcomes** | 3-5 |
| **Supplementary Figure 2: Leave-one-out analyses plots** | 6-8 |

**Supplementary Table 1: The risk of bias assessment of the included studies**

| The Revised Cochrane risk-of-bias tool for randomized trials for randomized controlled trials in the meta-analysis. | | | | | | | | | | | | | | |
| --- | --- | --- | --- | --- | --- | --- | --- | --- | --- | --- | --- | --- | --- | --- |
| Author, year | Bias arising from the randomization process | | Bias due to deviations from intended interventions | | Bias due to missing data | | | Bias in measurement of outcomes | | Bias in selection of the reported result | | low/moderate/serious / critical | | |
| Santer 2020 (MIDAS) | Low | | Low | | Low | | | Low | | Low | | Low | | |
| The Newcastle-Ottawa Scale for assessing the quality of nonrandomized studies in the meta-analysis. | | | | | | | | | | | | | | |
|  | Selection | | | | | | | | Outcome | | | | | |
| Study | Representative nest of the exposed cohort | Selection of the non-exposed cohort | | Ascertainment of exposure | | Outcome not present at baseline | Comparability of the cohort | | Assessment of outcome | | Enough follow up duration | | Adequate follow-up | Total score |
| Liu 2010 | * | * | | * | | * | * | | NA | | * | | NA | 6 |
| Poveromo  2016 | * | * | | * | | * | * | | * | | * | | * | 8 |
| Whitson  2016 | * | * | | * | | * | * | | * | | * | | * | 8 |
| Roach 2017 | * | * | | * | | * | NA | | NA | | * | | NA | 5 |
| Fiorenza 2019 | * | * | | * | | * | * | | NA | | * | | NA | 6 |
| Nadhim 2019 | * | * | | * | | * | NA | | NA | | * | | NA | 5 |
| Hailu 2020 | * | * | | * | | * | * | | NA | | * | | NA | 6 |
| Tremblay 2020 | * | * | | * | | * | * | | * | | * | | * | 8 |

Each asterisk represents one star in the Newcastle-Ottawa Scaling System (NOS). The maximum stars are 2 for comparability and 1 are for all other categories. Each star counts towards the total score. Score of 5 to 6 considered as moderate quality and 7 to 9 as high quality.

Abbreviation: NA: not available.

**Supplementary Figure 1-A: Baujat plot of mean duration of ICU length of stay**


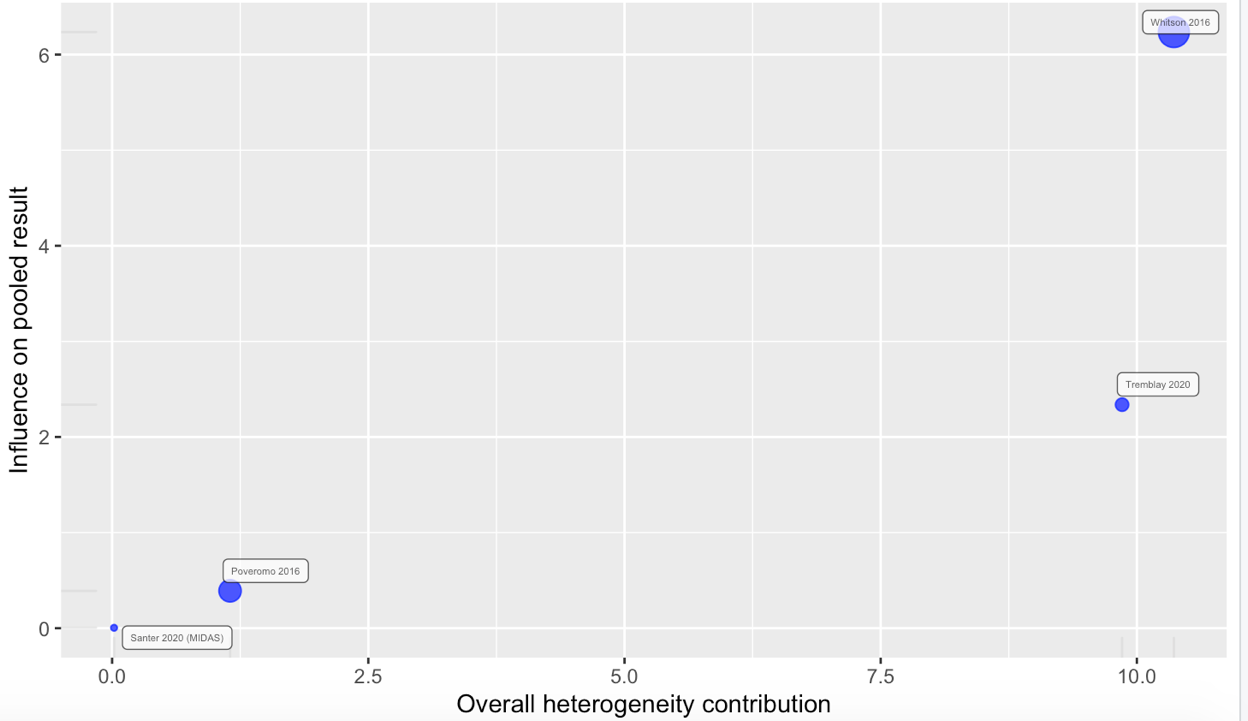


**Supplementary Figure 1-B: Baujat plot of mean duration of hospital length of stay**

**
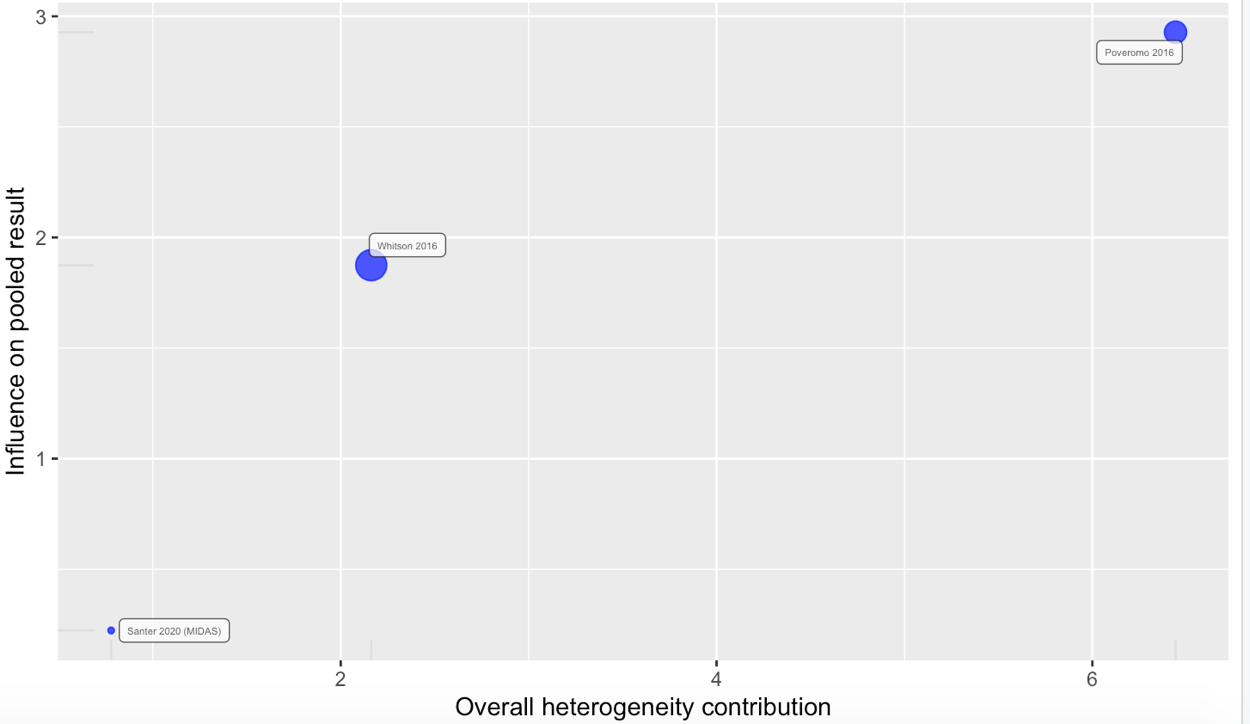
**

**Supplementary Figure 1-C: Baujat plot of in-hospital mortality**


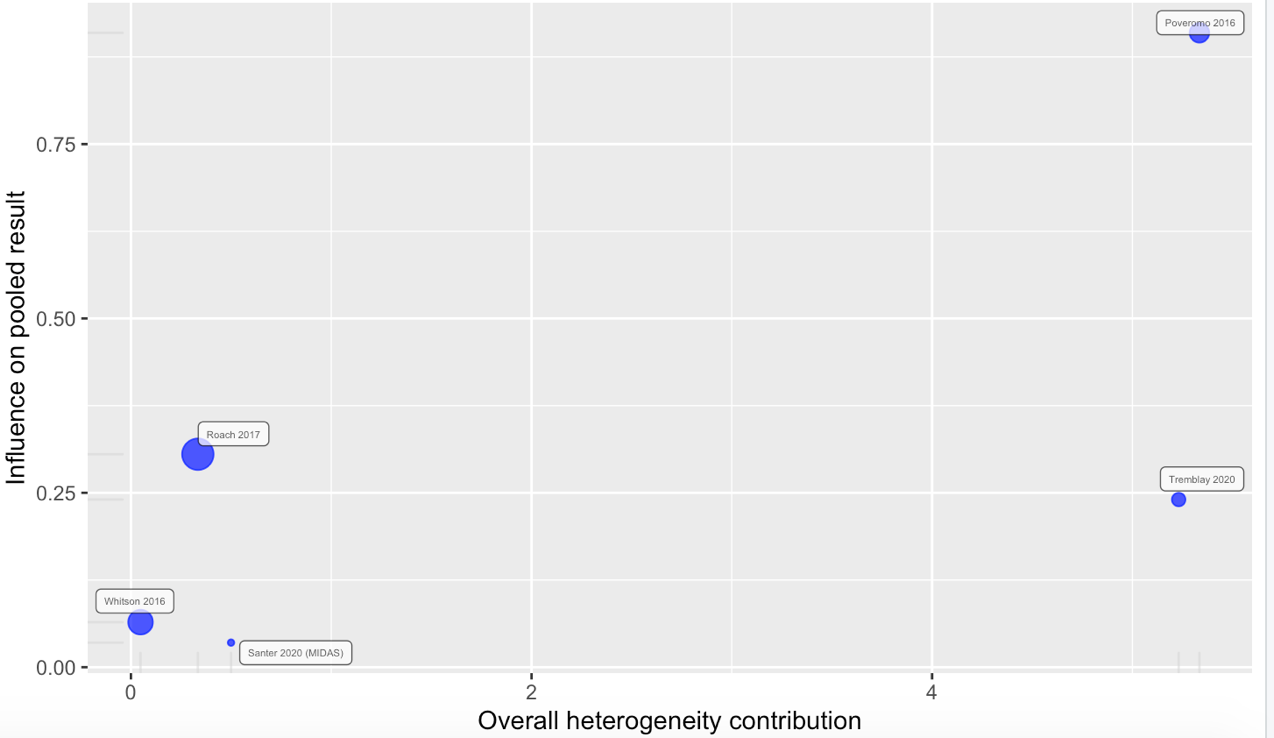


**Supplementary Figure 1-D: Baujat plot of intravenous vasopressors reinstitutions**


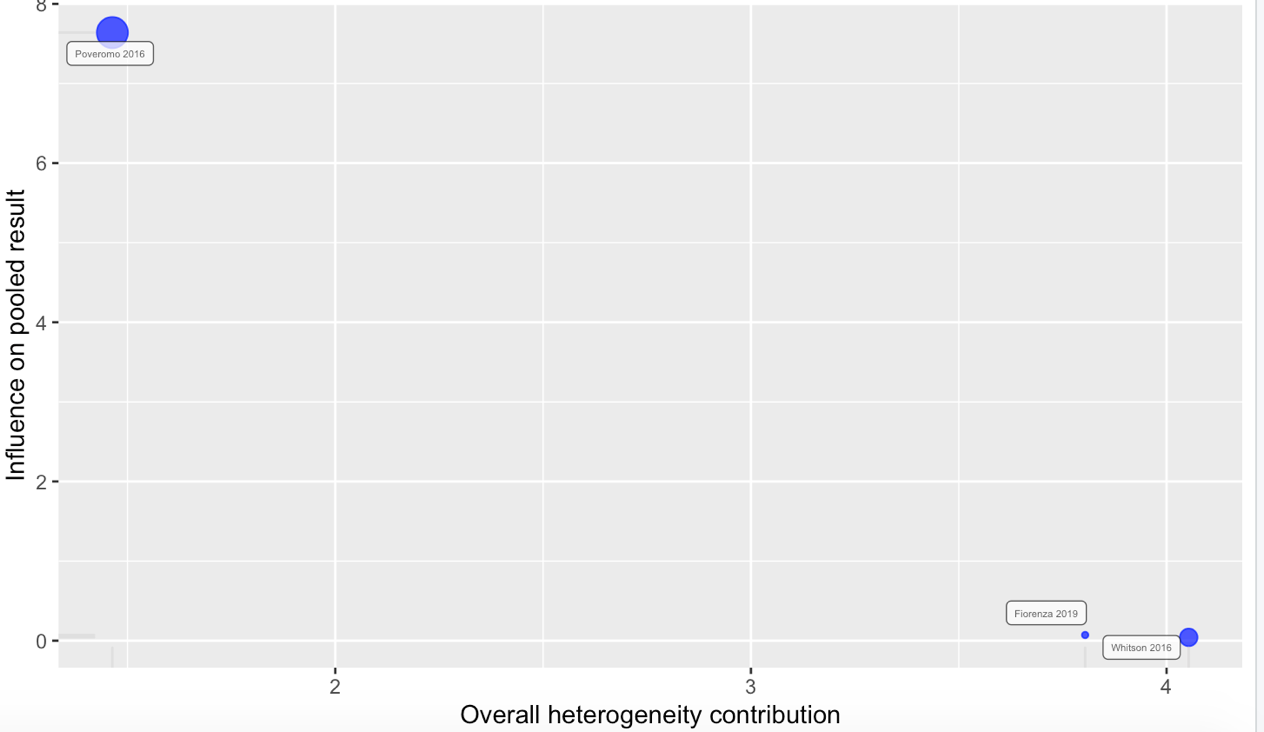


**Supplementary Figure 1-E: Baujat plot lot of ICU readmission**


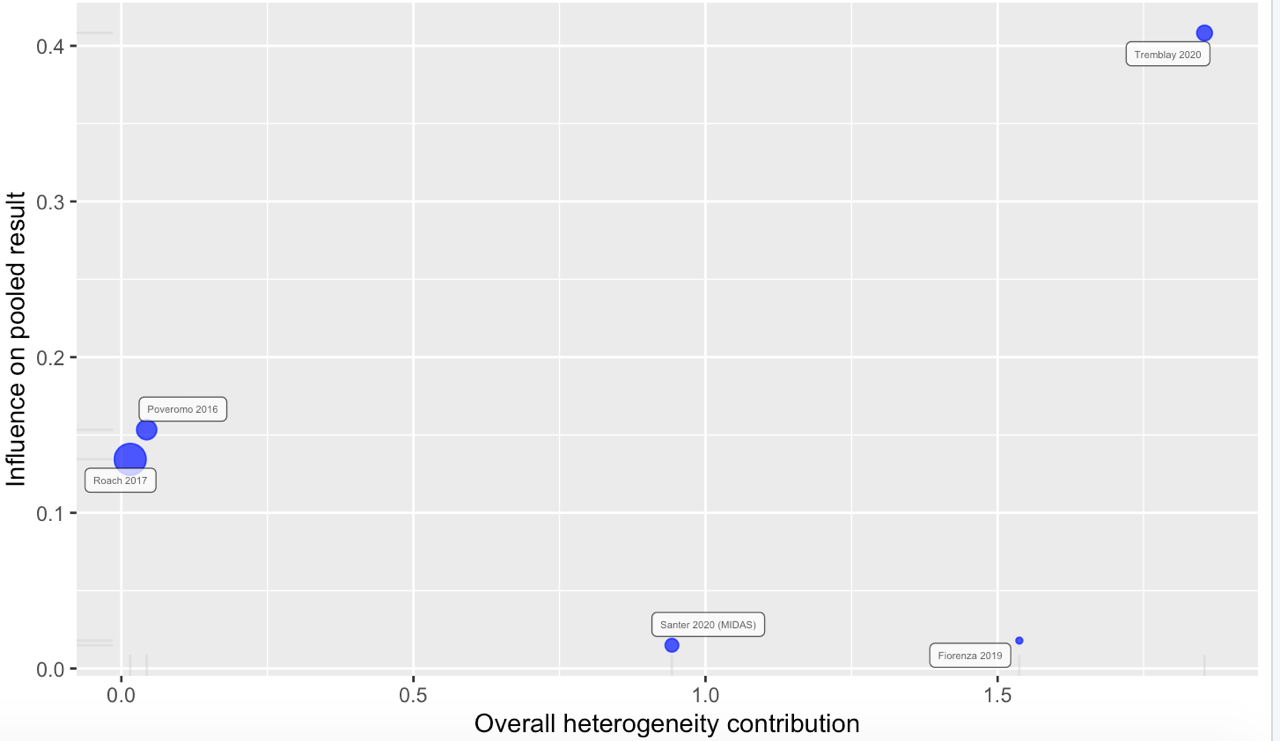

**Supplementary Figure 1-F: Baujat plot of bradycardia**


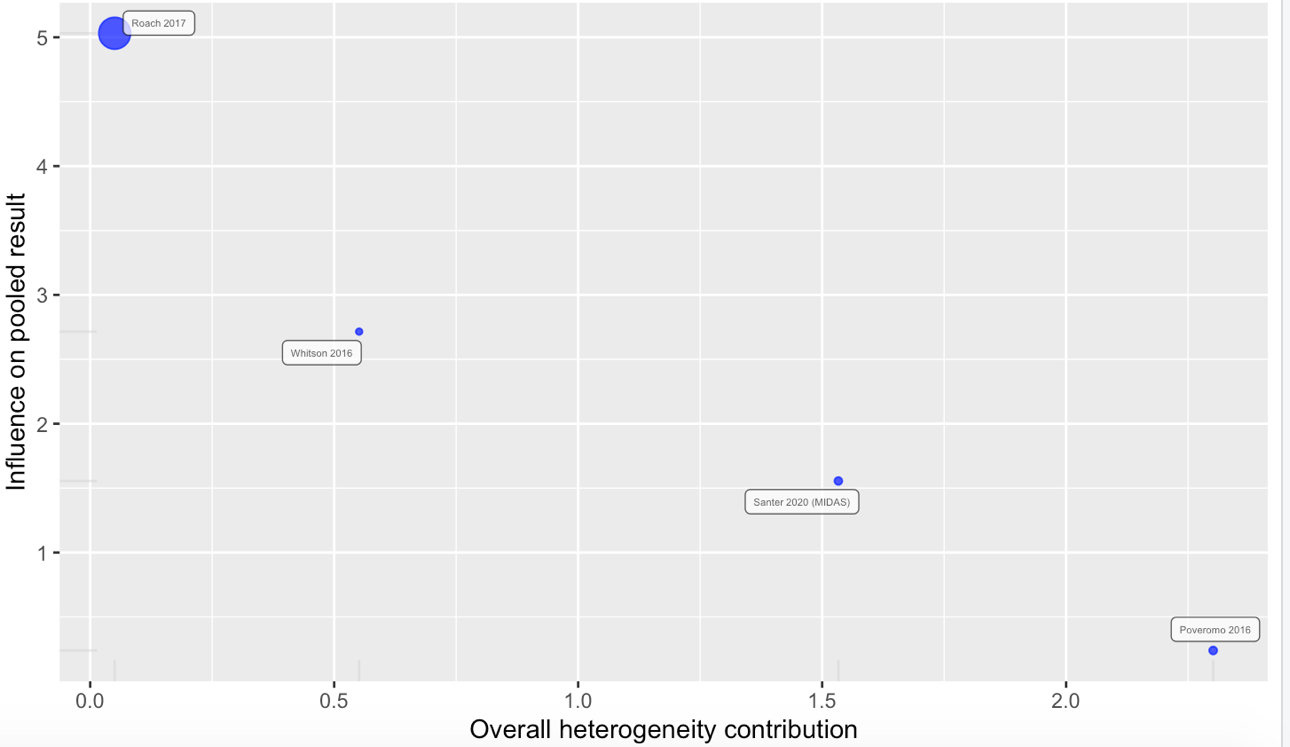


**Supplementary Figure 2-A: Leave-one out analysis of mean duration of ICU length of stay**


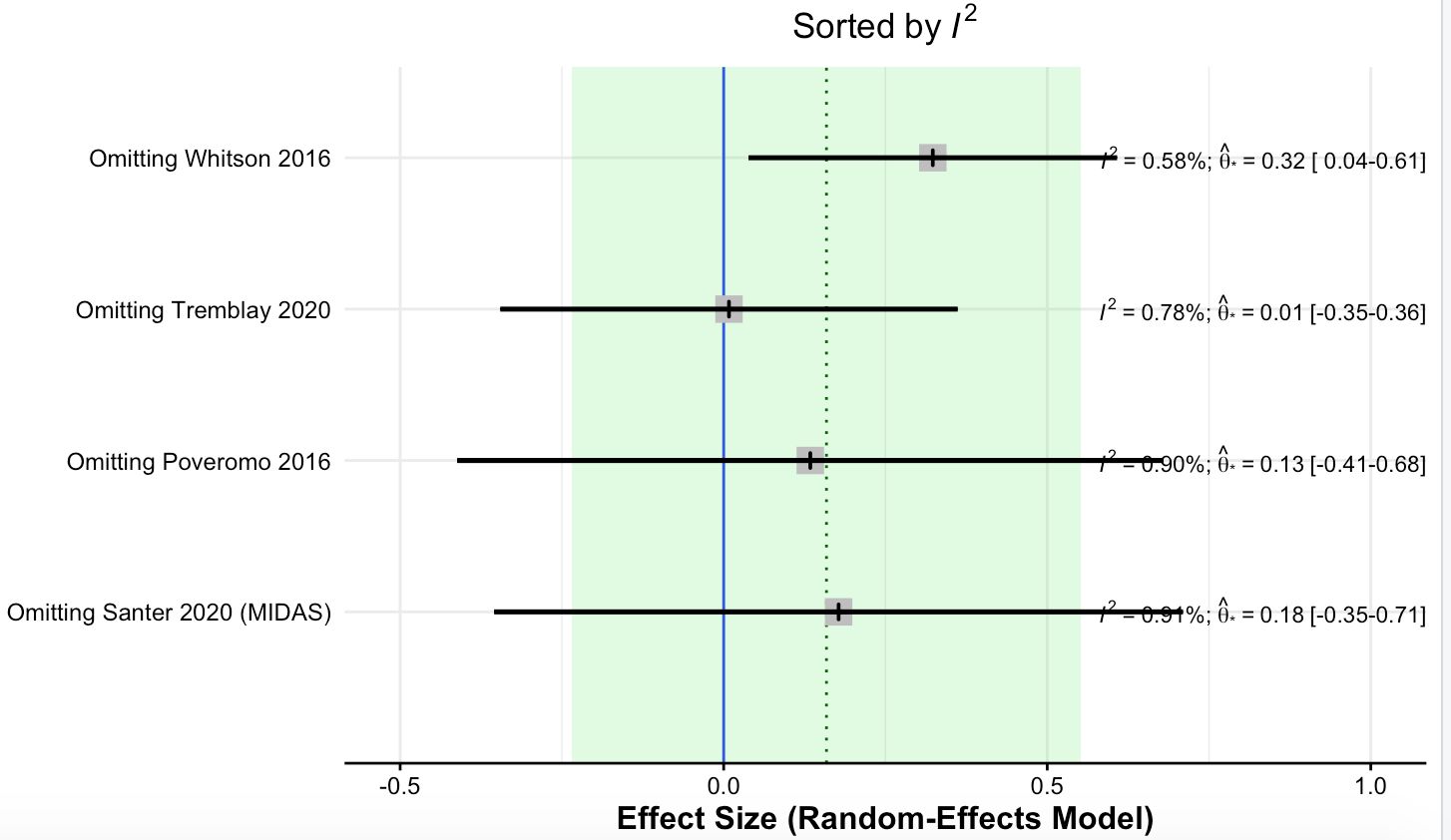


**Supplementary Figure 2-B: Leave-one out analysis of mean duration of hospital length of stay**


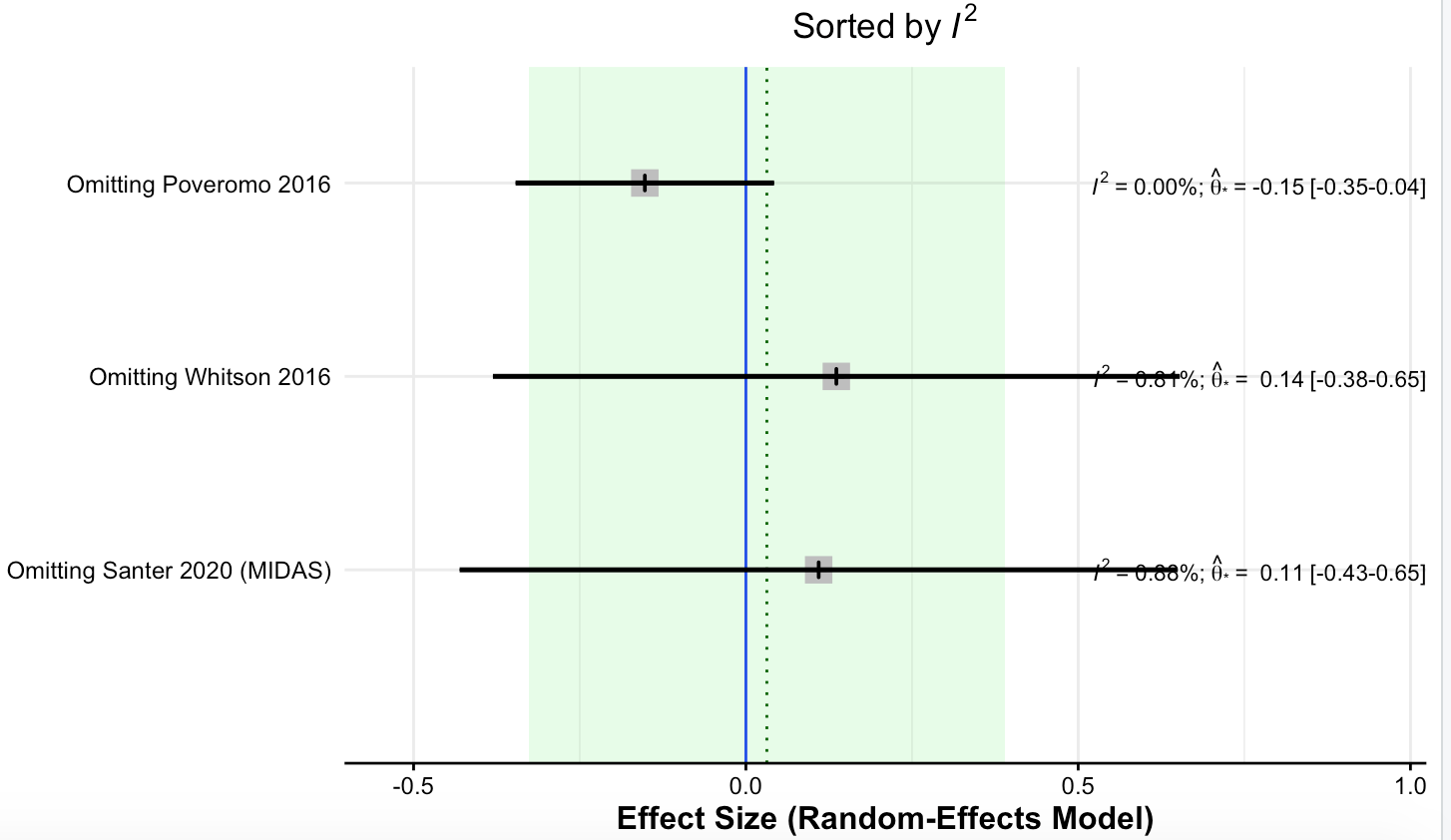


**Supplementary Figure 2-C: Leave-one out analysis of in-hospital mortality**


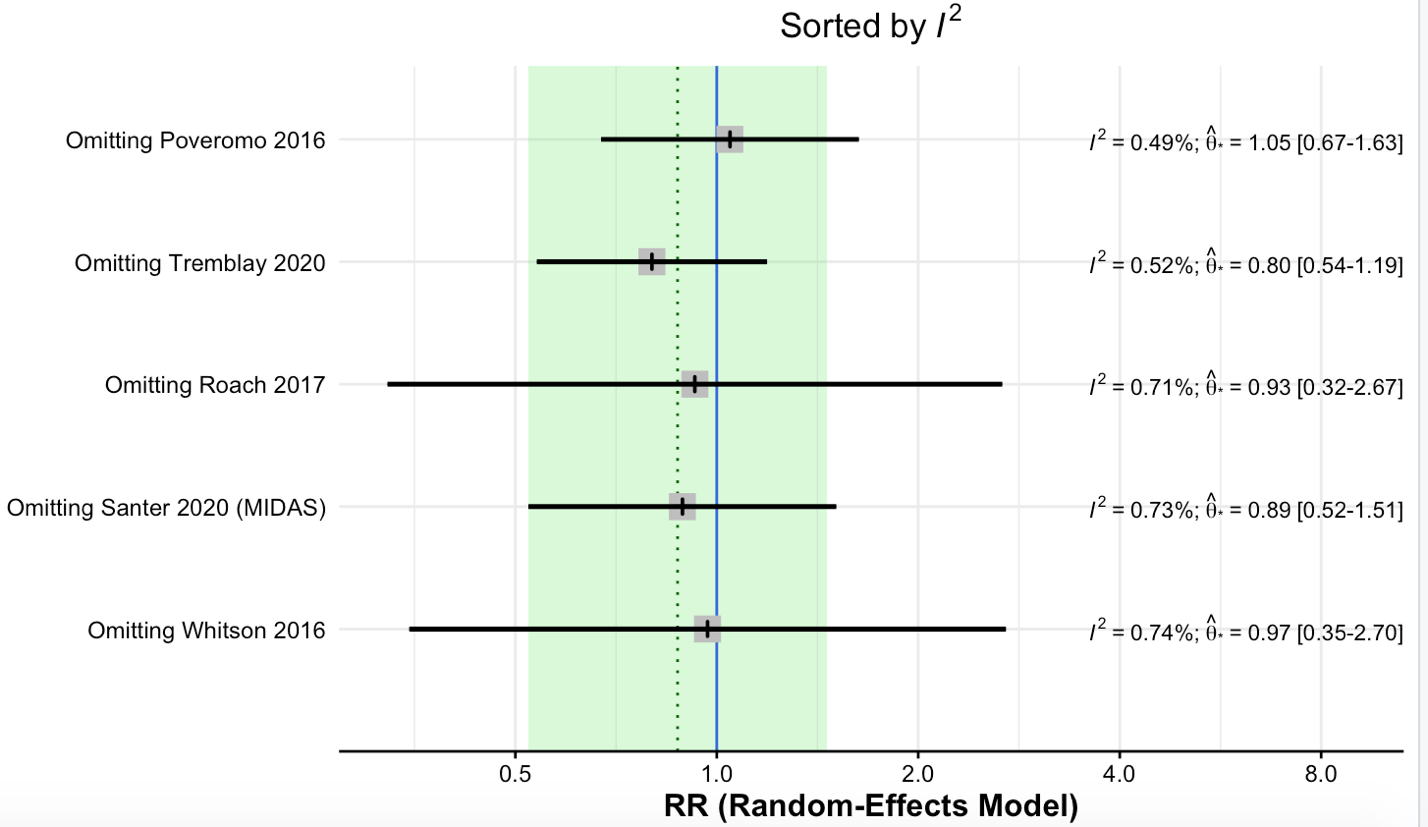
 **Supplementary Figure 2-D: Leave-one out analysis of intravenous vasopressors reinstitutions**


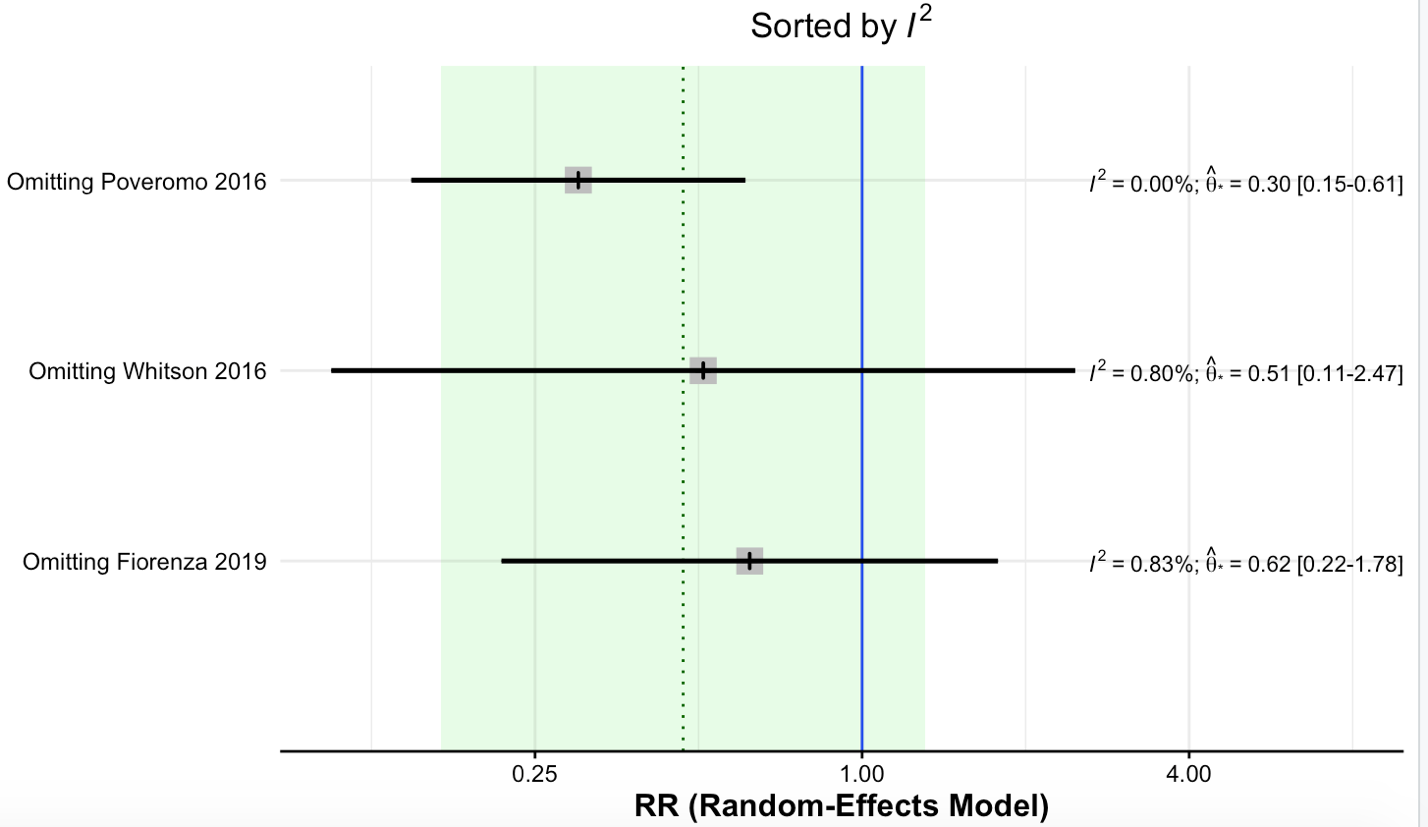


**Supplementary Figure 2-E: Leave-one out analysis of ICU readmission**


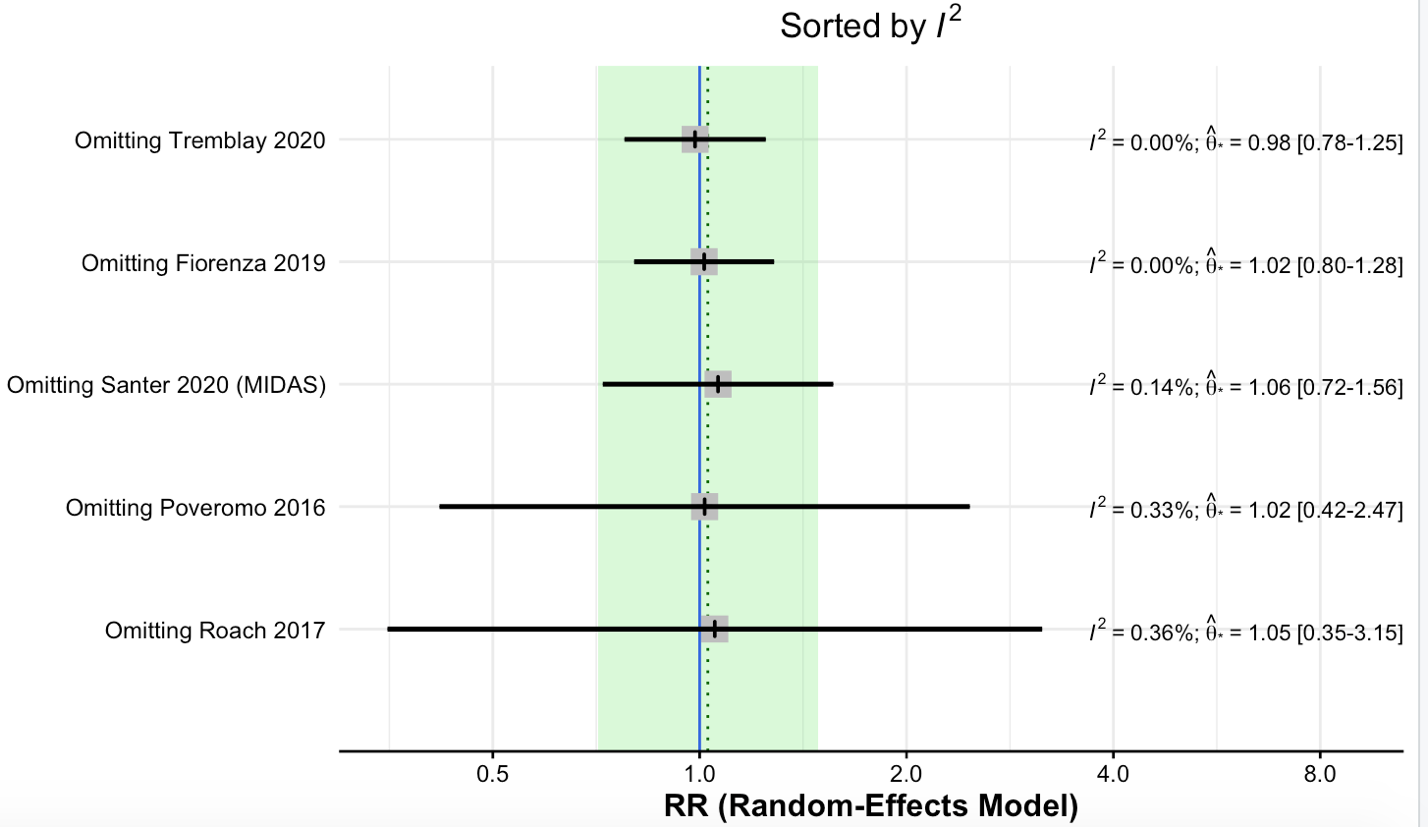


**Supplementary Figure 2-E: Leave-one out analysis of bradycardia**


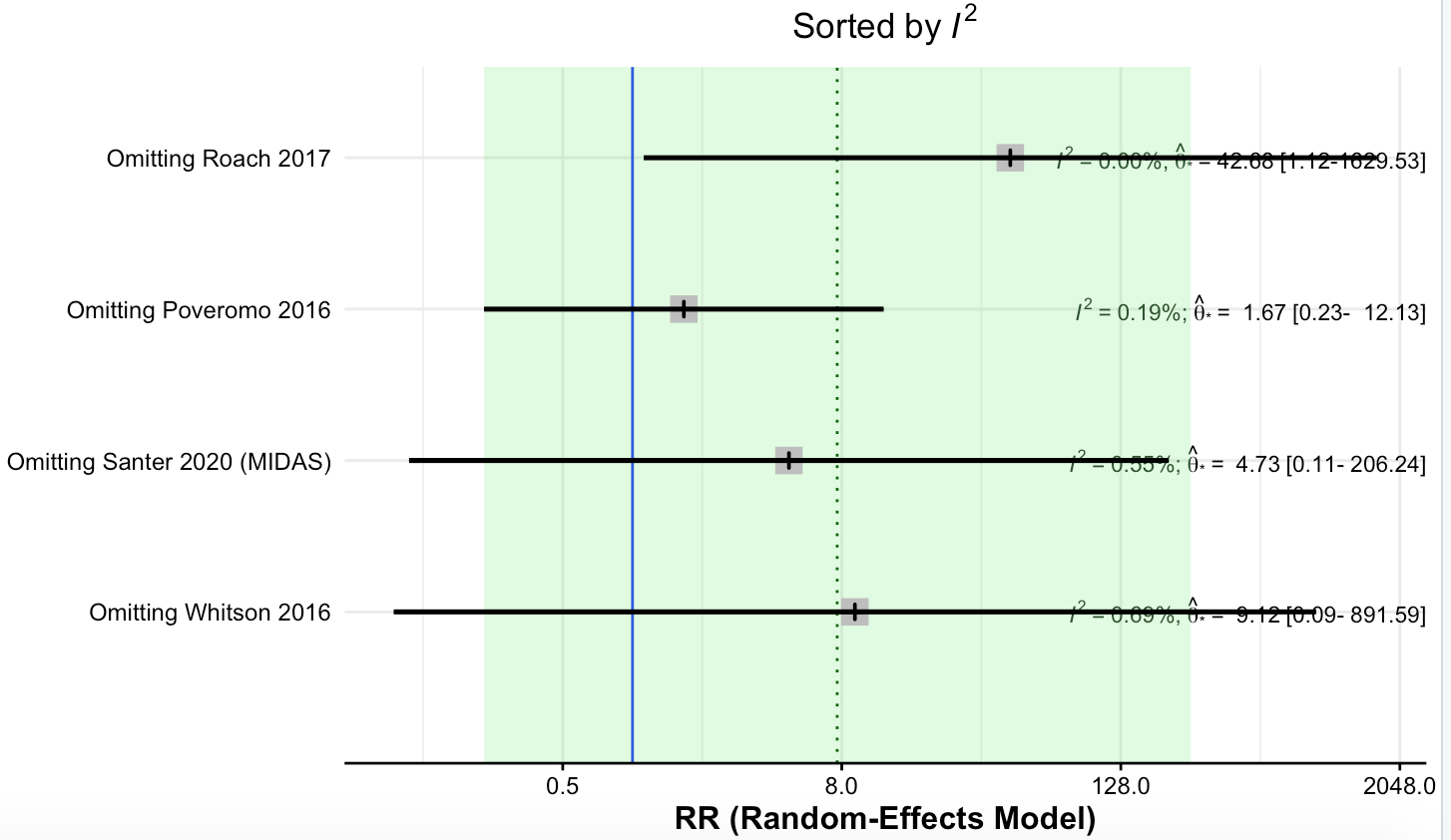

Supplement: Supplementary Materials — The supplementary material has detailed risk of bias and heterogeneity assessment, in addition to sensitivity analyses. [file 5588483.f1.docx]
